# Impact of Rifaximin on the Frequency and Characteristics of Spontaneous Bacterial Peritonitis in Patients with Liver Cirrhosis and Ascites

**DOI:** 10.1371/journal.pone.0093909

**Published:** 2014-04-08

**Authors:** Philipp Lutz, Marijo Parcina, Isabelle Bekeredjian-Ding, Hans Dieter Nischalke, Jacob Nattermann, Tilman Sauerbruch, Achim Hoerauf, Christian P. Strassburg, Ulrich Spengler

**Affiliations:** 1 Department of Internal Medicine I, University of Bonn, Bonn, Germany; 2 Institute for Medical Microbiology, Immunology and Parasitology, University of Bonn, Bonn, Germany; 3 German Center for Infection Research; University of Sydney, Australia

## Abstract

**Background:**

Rifaximin is a non-absorbable antibiotic used to prevent relapses of hepatic encephalopathy which may also be a candidate for prophylaxis of spontaneous bacterial peritonitis (SBP).

**Aim:**

To detect the impact of rifaximin on the occurrence and characteristics of SBP.

**Methods:**

We prospectively studied all hospitalized patients that underwent a diagnostic paracentesis in our department from March 2012 to April 2013 for SBP and recorded all clinical data including type of SBP prophylaxis, prior use of rifaximin, concomitant complications of cirrhosis, as well as laboratory results and bacteriological findings. Patients were divided into the following three groups: no antibiotic prophylaxis, prophylaxis with rifaximin or with systemically absorbed antibiotic prophylaxis.

**Results:**

Our study cohort comprised 152 patients with advanced liver cirrhosis, 32 of whom developed SBP during the study period. As expected, our study groups differed regarding a history of hepatic encephalopathy and SBP before inclusion into the study. None of the 17 patients on systemic antibiotic prophylaxis developed SBP while 8/27 patients on rifaximin and 24/108 without prophylaxis had SBP (p = 0.02 and p = 0.04 versus systemic antibiotics, respectively). In general, episodes of SBP were similar for patients treated with rifaximin and those without any prophylaxis. However, *Escherichia coli* and *enterococci* were dominant in the ascites of patients without any prophylaxis, while mostly *klebsiella* species were recovered from the ascites samples in the rifaximin group.

**Conclusion:**

Rifaximin pretreatment did not lead to a reduction of SBP occurrence in hospitalized patients with advanced liver disease. However, the bacterial species causing SBP were changed by rifaximin.

## Introduction

Spontaneous bacterial peritonitis (SBP) is a distinct form of infectious peritonitis occurring in patients with advanced liver cirrhosis and ascites [1]. Mortality of patients with SBP is high, with an in-hospital mortality of about 30% [2]. Recurrence of SBP is common [3], but can be prevented by secondary prophylaxis with systemic antibiotics [4].

Hepatic encephalopathy (HE) is considered to result from inadequate detoxification of intestinal toxins produced by intestinal bacteria. Also pro-inflammatory cytokines and formation of reactive oxygen species (ROS) contribute to this neuropsychiatric syndrome [5], [6]. Prevention of recurrent HE is achieved by administering lactulose, which alters the composition of intestinal bacteria. Recently, rifaximin, a non-absorbable antibiotic, has been introduced as a novel agent to prevent recurrent HE [7], [8].

Rifaximin has a broad spectrum of antibacterial activity. Concentrations in the stool are high, while absorption into the systemic circulation is negligible [9]. It has thus been proposed as an oral candidate antibiotic to prevent SBP in the absence of systemic side effects [1]. In mice, rifaximin has been demonstrated to reduce the progression of lipopolysaccharide-mediated fibrosis, but failed to prevent bacterial translocation [10], [11]. Therefore, it is undecided whether HE prophylaxis with rifaximin can reliably prevent SBP.

To clarify the impact of rifaximin on the frequency and features of SBP in cirrhotic patients with ascites, we prospectively evaluated all patients receiving paracentesis between March 2012 and April 2013 with respect to the presence of SBP and concomitant use of antimicrobial prophylaxis with rifaximin versus systemically absorbed antibiotics.

## Patients and Methods

### Ethics Statement

Written informed consent was obtained prior to patient recruitment and the study was approved by the local ethic committee of Bonn University Medical Center.

### Patients

We prospectively studied all patients with liver cirrhosis receiving a diagnostic paracentesis in the Department of Internal Medicine I of the University Bonn from March 2012 to the first week of April 2013 with respect to the presence of SBP. Time of inclusion was the time of first paracentesis during the study period. All patients with ascites due to liver cirrhosis above 17 years of age were included. Exclusion criteria were non-cirrhotic ascites (e.g. malignant ascites), age below 18 years, combined intake of both rifaximin and systemic antibiotic prophylaxis or presence of a permanent peritoneal catheter. The patients were stratified into 3 groups according to the type of prophylactic antibiotic treatment at the time of paracentesis. Group 1 comprised all patients without prophylaxis, group 2 all patients receiving rifaximin and group 3 all patients with systemically absorbed antibiotic prophylaxis that was given as primary or secondary SBP prophylaxis according to international guidelines [12]. Rifaximin was given 400 mg tid. A diagnostic paracentesis was performed whenever deemed necessary by the treating clinician on the basis of current guidelines [12].

Diagnosis of liver cirrhosis was based on complications of portal hypertension (oesophageal varices, splenomegaly and ascites), corresponding ultrasound and standard laboratory findings or liver biopsy, where available. Age, sex, etiology of cirrhosis, Child-Pugh stages, model for end-stage liver disease (MELD) scores, standard laboratory parameters, complications of cirrhosis (SBP, hepatocellular carcinoma, gastrointestinal bleeding, HE, number of hospital admissions during the three months previous to inclusion into the study), concomitant medication (rifaximin, systemic prophylactic antibiotics, beta blockers, proton pump inhibitors, lactulose, albumin substitution, diuretics and alpha blockers/vasopressin analogues) and, in the case of SBP, presence of indwelling catheters were recorded. SBP was diagnosed according to international guidelines if the polymorphonuclear leukocyte (PMN) cell count in the ascites exceeded 250/μl in the absence of other causes of peritonitis [12]. HE was assessed clinically and other causes of neuropsychiatric symptoms were excluded [13]. Rifaximin was given if patients had a second episode of HE of at least grade 2 according to the West Haven classification [13] that lead to hospitalization or significant impairment of daily activities.

Bacteria were classified as multi-resistant if they were methicillin-resistant *Staphylococcus aures*, vancomycin-resistant *enterococci* or Gram-negative bacteria resistant to at least three out of four of the following classes of antibiotics: penicillins, cephalosporins, carbapenems and quinolones.

### Methods

Albumin, bilirubin, creatinine, C-reactive protein, INR, sodium and total blood count were measured in the serum with standard procedures. Differential leukocyte counts, albumin and total protein were determined in the ascites.

Ten mL of the ascitic fluid were delivered into aerobic and anaerobic blood culture bottles (BD BACTEC, Becton Dickinson Heidelberg, Germany) and incubated for a maximum of 5 days in a Bactec FX blood culture system (Becton Dickinson) for microbial studies.

### Follow-Up

The patients had follow-up examinations at the time of discharge from the hospital and on every occasion when they presented again at our department thereafter for a maximum of 16 weeks. The median observation time was 3 weeks for patients without any prophylaxis, 4 weeks for patients treated by systemic prophylaxis and 4 weeks for patients treated with rifaximin (p = 0.4).

### Statistical Analysis

Data are reported as median and range, if not stated otherwise. Statistical analyses were performed with IBM SPSS Statistics software version 21 (IBM, New York, USA). For the analysis of quantitative data, the Wilcoxon-Mann-Whitney-U test and the Kruskal-Wallis-test were used as appropriate. Fisher's exact test was applied to qualitative data. A Kaplan-Meier plot was created and differences analysed with the log rank test for mortality after SBP and time-to-SBP in the different groups. P<0.05 was considered significant.

## Results

### Study Cohort

During the study period, 152 patients with liver cirrhosis underwent at least one diagnostic paracentesis in our department. 101 of these patients were male (66%), 61% had cirrhosis due to alcohol consumption. Advanced liver cirrhosis with Child-Pugh-stage B or C was common (149 patient, 98%). 32 patients (21%) were diagnosed with SBP.

Baseline characteristics of the patients in the predefined study groups are given in Table 1. The groups differed only by the history of complications reflecting the reasons for intake of different types of antibiotic prophylaxis. Since the approved indication for rifaximin is prevention of HE, all patients in group 2 had had at least two previous episode of HE (versus 8% in group 1 and 12% in group 3). In line with this, all patients in group 2 used lactulose (versus 55% in group 1 and 65% in group 3). HE is part of the Child-Pugh score, thus patients on rifaximin ranked significantly higher according to the Child-Pugh classification. Systemic antibiotic prophylaxis is frequently prescribed after a clinically apparent episode of SBP. In line with this, 89% of patients in group 3 had had a prior episode of SBP (versus 7% in group 1 and 15% in group 2). The remaining 11% of patients in group 3 without a previous SBP received antibiotic prophylaxis due to a history of bacterascites. Systemic antibiotic prophylaxis was done with quinolones (ciprofloxacin) in 96% of patients.

**Table 1:** Clinical and laboratory details of the study cohort according to the study groups.

		no prophylaxis	rifaximin	prophylaxis with systemic antibiotics	p
N		108	27	17	
Age [years]		60 (28 - 86)	61 (38 - 74)	62 (51 – 77)	0.75
Male sex	N (%)	74 (69%)	10 (60%)	17 (100%)	0.63
Etiology of cirrhosis	N (%)				0.12
Alcoholic		62 (57%)	20 (74%)	11 (65%)	
Viral		29 (27%)	5 (19%)	1 (6%)	
other		17 (16%)	2 (7%)	5 (29%)	
Child-Pugh-Stage A/B/C	(%)	1%/57%/43%	0/33%/67%	12%/47%/41%	**0.02**
MELD score		17 (6 – 41)	18 (8 – 41)	15 (6 – 26)	0.35
Bilirubin [μmol/L]		34 (5 – 616)	34 (9 – 496)	34 (5 – 137)	0.39
Creatinine [μmol/L]		106 (9 – 716)	133 (53 – 442)	124 (62 – 442)	0.33
INR		1.3 (1.0 – 2.5)	1.3 (1.1 – 2.8)	1.2 (1.0 – 1.7)	0.60
Albumin [g/L]		29 (11 – 48)	27 (17 – 44)	27 (15 – 42)	0.09
Ascites protein [g/L]		11 (2 – 44)	10 (3 – 31)	11 (2 – 43)	0.81
Proton pump inhibitors	N (%)	85 (79%)	24 (89%)	15 (94%)	0.32
Beta-Blocker	N (%)	48 (46%)	14 (52%)	5 (31%)	0.41
Lactulose	N (%)	59 (55%)	27 (100%)	11 (65%)	**0.001**
Albumin substitution	N (%)	108 (100%)	27 (100%)	17 (100%)	
Diuretics	N (%)	93 (86%)	13 (85%)	13 (77%)	0.59
Splanchnic vasoconstrictors (alpha blocker/vasopressin analogue)	N (%)	20 (19%)	7 (26%)	3 (18%)	0.65
Oral quinolones/cephalosporines	N (%)			16 (95%)/1 (4%)	
hospital admissions during previous three months	N (%)	1 (0 – 10)	2 (0 - 6)	2 (1 - 3)	0.49
Hepatocellular carcinoma	N (%)	25 (23%)	6 (22%)	1 (6%)	0.30
previous SBP	N (%)	8 (7%)	4 (15%)	15 (89%)	**<0.001**
previous HE	N (%)	9 (8%)	27 (100%)	2 (12%)	**<0.001**
Acute gastrointestinal bleeding	N (%)	10 (9%)	4 (15%)	1 (6%)	0.67

Data are given as patient numbers (percentage) or median (range).

Concerning laboratory parameters and the common complications of liver cirrhosis, there were no significant differences between the groups. Patients with previous HE did not have significantly higher rates of SBP during the study period in comparison to patients without previous HE (32% versus 18%; p = 0.11).

### Comparison of SBP Frequencies

**Figure 1:**
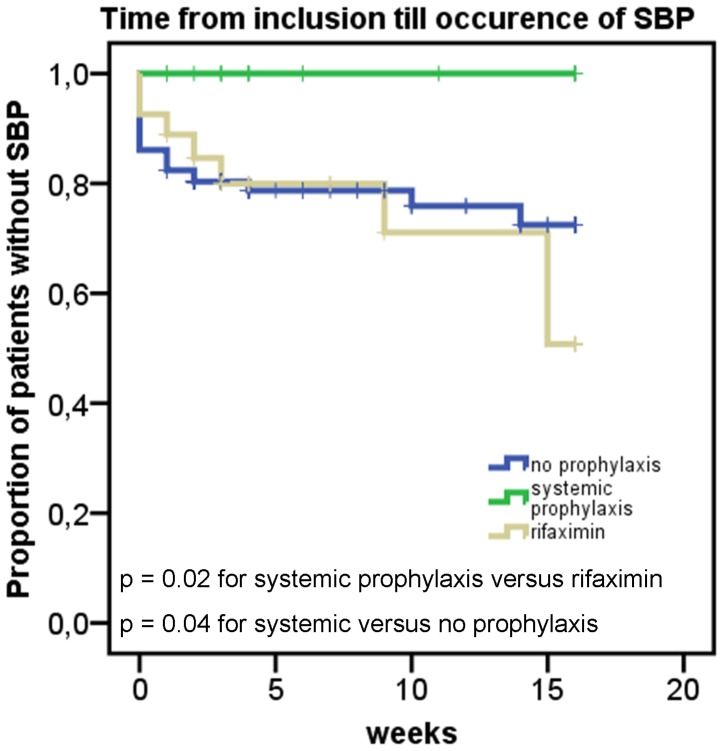
Time interval from study inclusion till occurrence of SBP. Kaplan-Meier-Plot of the time interval from study inclusion till occurrence of SBP or last observation. Statistical analysis with log rank test.

To assess the prophylactic efficacy of rifaximin versus systemic antibiotic prophylaxis, we compared the frequency of SBP in the different groups. SBP occurred in 24 patients in group 1 (22%), 8 patients (30%) in group 2 and no patient in group 3, which indicates a significant benefit of systemically absorbed antibiotic prophylaxis (p = 0.04 and p = 0.02 compared to no prophylaxis and prophylaxis with rifaximin, respectively). In order to adjust for differences in liver disease severity between patients in group 2 and 3, we excluded all patients with a MELD score above 26 from group 2. This lead to a SBP frequency of 6/23 patients in group 2 (26%), which was still significantly different from group 3 (p = 0.03). In addition, analysis of the time interval between inclusion into the study and occurrence of SBP (Fig. 1) indicated that systemic antibiotic prophylaxis was superior to rifaximin (p = 0.02) or no prophylaxis (p = 0.04). 15 patients in group 1 and 2 patients in group 2 had SBP at the first paracentesis during the study period.

### Characteristics of Patients with SBP

Next, we analysed the clinical and laboratory details of the patients who were diagnosed with SBP. As expected, patients with SBP had significantly higher serum levels of creatinine, INR, C-reactive protein, increased leukocyte counts in the blood and the ascites and higher MELD scores (Table 2). In line with the literature, patients with acute gastrointestinal bleeding developed SBP more frequently than those without acute bleeding (40% versus 19%, p = 0.09) [1].

**Table 2:** Statistically significant differences in laboratory parameters between patients with and without SBP.

	no SBP	SBP	p
N	120	32	
Creatinine [mg/dL]	1.1 (0.1 – 6.9)	1.9 (0.6 – 8.1)	**0.001**
INR	1.3 (1.0 – 2.8)	1.4 (1.0 – 2.7)	**0.018**
MELD score	16 (6 – 39)	21 (7 – 41)	**0.001**
CRP [mg/L]	21 (0.7 – 160)	57 (10 – 219)	**< 0.001**
Blood leukocyte count [G/L]	7.7 (1.6 – 40)	11.5 (0.6 – 37)	**0.001**
Ascites leukocyte count [G/L]	136 (23 – 1069)	1355 (476 – 24044)	**< 0.001**
Ascites PMN cell count [G/L]	18 (1 – 237)	1088 (256 – 23593)	**< 0.001**

Data are given as median (range).

Similar to the entire cohort, SBP patients who received rifaximin had more often a history of previous recurrent HE and higher Child-Pugh scores in comparison to SBP patients without rifaximin, reflecting that it is used to prevent further episode of HE. However, patients on rifaximin treatment did not differ regarding laboratory results, the presence of other complications of liver cirrhosis, nor the etiology of liver disease (Table 3).

**Table 3:** Characteristics of patients with SBP in correlation to the use of rifaximin.

		no prophylaxis	rifaximin	p
N		24	8	
Male sex	N (%)	16 (67%)	3 (38%)	0.22
Etiology of cirrhosis	N (%)			0.92
Alcoholic		16 (67%)	6 (75%)	
Viral		5 (21%)	1 (13%)	
Other		3 (12%)	1 (13%)	
Child-Pugh-Stage A/B/C	N (%)	9 (38%)/15 (63%)	8 (100%)	0.07
MELD score		22 (7 – 41)	20 (11 – 41)	0.85
Bilirubin [μmol/L]		38 (5 - 616)	44 (17 - 274)	0.48
Creatinine [μmol/L]		177 (53 – 707)	141 (62 – 362)	0.56
INR		1.5 (1.0 – 2.5)	1.4 (1.3 – 2.7)	0.78
Albumin [g/L]		29 (11 – 37)	27 (22 – 29)	0.16
Ascites total protein [g/L]		9 (3 – 42)	7 (4 – 18)	0.48
Proton pump inhibitor	N (%)	20 (84%)	8 (100%)	0.55
Beta-Blocker	N (%)	8 (35%)	5 (63%)	0.23
Renal replacement therapy	N (%)	1 (4%)	1 (13%)	0.44
Hepatocellular carcinoma	N (%)	4 (17%)	0	0.55
previous SBP	N (%)	6 (25%)	1 (13%)	0.65
previous HE	N (%)	5 (21%)	8 (100%)	**<0.0001**

Data are given as patient numbers (percentage) or median (range).

### Characteristics of SBP

The characteristics of SBP episodes between the groups were compared. Levels of inflammatory parameters (C-reactive protein, leukocyte counts in blood and ascites), nosocomial origin of infection, presence of indwelling catheters and the presence of acute gastrointestinal bleeding in the context of SBP were comparable between the groups. Since all patients had been in contact with the healthcare system during the last 3 months, no SBP was considered to be community acquired. The 30 day mortality did not differ significantly between patients taking rifaximin compared to patients without rifaximin (15% versus 32%, p = 0.42). This was also observed for the median survival (6 weeks versus 9 weeks, p = 0.27). However, the study was not sufficiently powered to detect such differences.

Rates of positive ascites cultures were comparable (Table 4). In addition, detection rates of bacteria resistant to third generation cephalosporins and of multi-resistant bacteria were not significantly different. However, the isolated bacterial species differed between patients with and without rifaximin pretreatment. While infections with *enterococci* and *Escherichia coli* accounted for 72% of positive ascites cultures in patients without any prophylaxis, none of these bacteria were identified in the ascites of patients taking rifaximin. In contrast, 75% of the micro-organisms detected in the ascites of patients treated with rifaximin were *klebsiella* species. This difference in the pattern of recovered microorganisms was statistically significant (Fisher's exact test, p = 0.01).

**Table 4:** Laboratory and microbiological features of SBP in patients with and without rifaximin pretreatment.

		no prophylaxis	Rifaximin	p
N		24	8	
C reactive protein [mg/dL]		59 (11 – 215)	47 (10 – 219)	0.31
Blood leukocyte count [G/L]		11.6 (0.6 – 37)	11.2 (7.0 – 23.7)	1.0
Ascites leukocyte count [G/L]		1343 (476 – 24044)	1416 (486 – 4890)	0.98
Ascites PMN cell count [G/L]		1089 (294 – 23593)	1089 (256 – 3679)	1.0
Nosocomial infection	N (%)	13 (54%)	3 (38%)	0.69
Indwelling catheter	N (%)	7 (29%)	2 (25%)	1.0
Acute gastrointestinal bleeding	N (%)	2 (8%)	1 (13%)	1.0
Positive ascites cultures	N (%)	11 (46%)	4 (50%)	1.0
Gram positive infections	N (%)	5 (45%)	0	0.23
Identified bacteria:	N (%)			**0.01**
* Enterobacter cloacae*		1 (9%)		
* Enterococcus faecalis/faecium*		3 (27%)		
* Escherichia coli*		5 (45%)		
* Serratia marcescens*		1 (9%)		
* Staphylococcus aureus*		1 (9%)		
* Klebsiella pneumoniae/oxytoca*			3 (75%)	
* Pasteurella multocida*			1 (25%)	
Resistance to third generation cephalosporins	N (%)	5 (46%)	1 (25%)	0.6
Multiresistant bacteria	N (%)	1 (9%)	1 (25%)	0.48

Data are given as patient numbers (percentage) or median (range).

## Discussion

Rifaximin, a non-absorbable antibiotic, has been licensed for the prevention of relapsing HE [7]. In addition, due to its broad intestinal antibacterial activity, it is a candidate for the prevention of SBP, which is attributed to intestinal bacterial transmigration [1]. Therefore, we prospectively studied the impact of rifaximin co-medication on SBP in 152 patients undergoing diagnostic paracentesis in our department.

Comparing the efficacy of rifaximin and systemic antibiotics as SBP prophylaxis in our cohort, we found a significantly lower rate of SBP in patients treated with systemic antibiotic prophylaxis, while SBP rates in patients with no prophylactic treatment and in patients taking rifaximin were comparable. In contrast, Hanouneh et al. recently reported a retrospective study of 404 cirrhotic patients with HE where rifaximin effectively prevented SBP [14]. However, the authors did not compare rifaximin to systemic prophylaxis, which is an established clinical standard to prevent recurrent SBP. Furthermore, that study excluded all patients with a high risk for SBP. Another difference to our study is that the authors only found culture-negative SBP in their patients on rifaximin. Overall, the sensitivity of bacteriological culture was lower in their study (30%) compared to our study (47%) and the average sensitivity reported in the literature (∼40%) [1], [15], [16]. Of note, culture positive SBP has been associated with an increased mortality [17]. Another small case control study [18] reported a preventive effect of rifaximin on SBP in a cohort of patients with decompensated cirrhosis. However, this study only included patients who had shown a decrease in the hepatic venous pressure gradient after an initial course of rifaximin. The authors found a 5-year cumulative survival of 61%. This is remarkable, considering that 5-year mortality in patients with decompensated liver cirrhosis has been reported to be up to 85% [19]. Taken together, these data indicate that there may be a subgroup of cirrhotic patients that benefits from rifaximin. However, our findings suggest that, overall, systemic antibiotic prophylaxis against SBP is more effective than rifaximin and should be considered as the standard of care in patients with advanced cirrhosis and a high risk of SBP.

Rifaximin is a candidate for SBP prevention because it shows broad intestinal antibacterial activity without systemic side effects and because SBP is thought to occur from bacterial translocation. A possible explanation for episodes of SBP during rifaximin treatment could be resistance to rifaximin. This issue is controversial and not easy to resolve. Some studies reported a slow development and rapid disappearance of resistance to rifaximin [7], [20], [21]. In contrast, more recent studies found persistently high rates of resistance in ileal *E. coli* [22] and in *staphylococci* [23]. The definition of resistance to rifaximin is difficult, since no data on the intestinal drug concentration are available to define a cut-off for minimal inhibitory concentrations. Fecal levels of rifaximin are very high [24], [25], but do not necessarily reflect the intra-luminal situation in cirrhotic patients. Apart from one case of *Pasteurella multocida*, which is a rare cause of SBP transmitted from pets [26], we only found *klebsiella species* isolates in our patients with SBP after rifaximin pretreatment. Thus far, only one additional case of culture-positive SBP during treatment with rifaximin has been reported in the literature [18], which was caused by *E. coli*. Interestingly, an increased minimal inhibitory concentration (MIC) of *klebsiella* species to rifaximin in comparison to the MIC of most Gram-positive bacteria has been described [25]. Thus, differential susceptibility of bacterial species to rifaximin might be one explanation why rifaximin does not fully prevent SBP.

Resistance patterns to systemic antibiotics were similar between the two groups. This is important for the initial treatment of patients with liver cirrhosis and suspected bacterial infection, since SBP is the most common source of bacterial infections in these patients [27] and emerging resistance leads to failure of empiric treatment [28]. In line with our results, another recent study did not find any impact of rifaximin on the development of bacterial resistance in cirrhotic patients [16]. However, this study did not evaluate the effect of rifaximin on SBP separately.

Given that immune defects are associated with liver cirrhosis and that rifaximin lacks systemic effects, a general reduction of intestinal bacterial loads by rifaximin may suffice to significantly reduce toxin production and to prevent HE, but may not be sufficient for SBP prevention if mucosal translocation of small amounts of bacteria still occurs [27]. This hypothesis is supported by a recent study in cirrhotic patients demonstrating that rifaximin treatment changed the pattern of metabolites produced by the intestinal bacteria rather than the quantity of bacteria [29]. In line with this concept, prophylaxis with systemic, but not local antibiotics reduced SBP rates significantly in our cohort.

Another possible explanation for the occurrence of SBP under treatment with rifaximin is that direct bacterial translocation from the intestine might be only one of several routes of infection, as supported by analysis of bacterial DNA in ascites [30]. Bacteremia without an apparent source is common in cirrhosis [31]. In general, the risk of bacteremia is related to oral health and daily oral activities like tooth brushing [32]. Interestingly, both *klebsiella* species and *Pasteurella multocida* have been described as part of the oral human microbiome [33], [34], [35]. In addition, k*lebsiella* and p*asteurella* are known to cause SBP [36], [26]. Oral translocation of bacteria may therefore represent another possible source of SBP in patients treated with rifaximin.

The most important limitation of our study is that it was not a randomized, placebo controlled trial, but a prospective longitudinal observational study. In this type of study, a selection bias cannot be ruled out completely. Patients with HE may be more prone to further complications of liver cirrhosis than patients without HE. Given the high mortality of SBP and the excellent preventive efficacy of prophylactic systemic antibiotics, ethical concerns must be raised against a randomized controlled trial withholding systemic prophylaxis to the experimental arm without previous data indicating a potential benefit of rifaximin. At presence, such data are not available. Thus our investigation was an explorative study. The size of possible effects was unknown, so that a-priori power analysis was not possible. An important difference between group 2 and 3 is that for most patients in group 2, rifaximin was a primary prophylaxis against SBP while nearly all patients in group 3 took antibiotics as secondary prophylaxis. Secondary in contrast to primary prophylaxis can be targeted to the microorganism that caused SBP. However, secondary prophylaxis was based on quinolones which are also the first choice of systemic antibiotics in primary prophylaxis [12]. The responsible microorganism was isolated only in 47% of SBP cases, limiting the possibility to target secondary prophylaxis. Finally, the risk to acquire SBP is in part genetically determined [37], [38], which might compensate for the advantages of secondary versus primary prophylaxis.

Our results suggest that rifaximin should not generally replace systemically absorbed antibiotics for SBP prophylaxis in patients at high risk for SBP and with recurrent hospitalisations. SBP is a complication of advanced liver disease and our cohort is typical for patients with advanced cirrhosis. However, it remains open whether our findings can be extrapolated to patients at low risk and with less severe liver disease. A further limitation is the fact that we did not measure rifaximin levels in patient stool to exclude non-adherence with drug therapy. However, the observed clinical improvement of HE suggests good adherence in the studied patient cohort. In addition, the biological intestinal half time of rifaximin is several days [9], [39] and consequently even the omission of one or two dosages would not result in insufficient drug levels. Future studies on the effects of rifaximin on SBP should include assessment of bacterial resistance to rifaximin, which is complicated by the unavailability of commercially available resistance tests or standardized testing procedures with normal values.

In summary, our data indicate that rifaximin cannot prevent SBP reliably in patients with advanced liver disease. SBP prophylaxis continues to rely on systemic antibiotics, at least until controlled studies have clarified which subgroup of patients with cirrhosis can benefit from rifaximin rather than from systemically absorbed antibiotics.
